# Paraphilic Disorder and Gender Dysphoria in a Case with High-Functioning Autism Spectrum Disorder

**DOI:** 10.1192/j.eurpsy.2022.1176

**Published:** 2022-09-01

**Authors:** U. Altunoz, J. Krueger, M. Ziegenbein

**Affiliations:** 1 Wahrendorff Clinic, Centre Of Transcultural Psychiatry & Psychotherapy, Sehnde, Germany; 2 Hannover Medical School, Research Group For Social And Transcultural Psychiatry & Psychotherapy, Hannover, Germany; 3 Wahrendorff Clinic, Department Of Psychiatry & Psychotherapy, Sehnde, Germany

**Keywords:** Gender Dysphoria, Autismus Spectrum Disorder, paraphilic disorders

## Abstract

**Introduction:**

Autism spectrum disorder (ASD) is a neurodevelopmental disorder characterized with difficulties in social interaction/communication, restricted interests, and repetitive behaviors. Sexual issues such as paraphilic behaviors in ASD have gained attention in recent years, however there is still a great paucity of research regarding this issue.

**Objectives:**

The aim of this presentation is to draw attention to a crucial dimension through a case of ASD with paraphilic disorder (pedophilic tendencies) and gender dysphoria.

**Methods:**

One case from an inpatient unit of a psychiatric clinic in Lower Saxony, Germany will be reported.

**Results:**

Case: An 18-year-old male was referred to our acute psychiatric ward due to suicidal thoughts and other depressive symptoms. In further examination, he stated that he had pedophilic phantasies which he was trying to satisfy by using child pornography in the darknet. He had never been involved in any sexual relationship with a child and described this behavior as an addiction that he wanted to get rid of. Detailed psychiatric examination and developmental history yielded the diagnosis of high-functioning ASD. The compulsory paraphilic engagement is classified as a restrictive-repetitive interest in terms of ASD. In addition, the patient presented gender incongruence with moderate gender dysphoria, dressed in a skirt and wanted to be perceived and named rather gender-neutral, which was supported through the whole course.

**Conclusions:**

Through systemic understanding of the high-functioning ASD structure and complex symptomatology, socio- and psychotherapeutic approaches were implemented which yielded an apparent stabilization. The detailed therapeutic process in the light of the present literature will be discussed.

**Disclosure:**

No significant relationships.

